# An EEG dataset for handwriting imagery decoding of Chinese character strokes and Pinyin single vowels

**DOI:** 10.1038/s41597-026-06708-3

**Published:** 2026-02-02

**Authors:** Fan Wang, Yanxiao Chen, Peng Wang, Anmin Gong, Jiaping Xu, Yunfa Fu

**Affiliations:** 1 Faculty of Information Engineering and Automation, Kunming University of Science and Technology, Kunming, 650500 China; 2 Brain Cognition and Brain-computer Intelligence Integration Group, Kunming University of Science and Technology, Kunming, 650500 China; 3 School of Information Engineering, Chinese People’s Armed Police Force Engineering University, Xi’an, 710000 China

**Keywords:** Cognitive neuroscience, Brain-machine interface

## Abstract

Non-invasive EEG-based brain-computer interfaces (BCI) for handwriting imagery can support the restoration of fine writing abilities in individuals with motor impairments. However, the development of high-performance decoding algorithms is constrained by scarce training datasets. To address this, we present the first open EEG dataset dedicated to handwriting imagery. The dataset comprises 32-channel EEG recordings (sampled at 1000 Hz) from 21 healthy participants across two sessions separated by at least 24 hours. A dual-paradigm design captures multidimensional neural features: a Chinese character stroke imagery task (five basic strokes, 200 trials per session) and a Pinyin single-vowel imagery task (six vowels, 240 trials per session). After rigorous quality screening, 18,480 standardized trials are provided, ensuring completeness, reliability, and adherence to the Brain Imaging Data Structure (BIDS) standard. This dataset enables the development and evaluation of algorithms for non-invasive BCI and supports research on restoring writing-based communication in individuals with motor impairments.

## Background & Summary

Every year, traumatic brain injuries, stroke, and neurodegenerative diseases cause thousands of patients to lose handwriting and other communication abilities, severely impairing daily interactions^1–3^. Brain-computer interface (BCI) has shown considerable potential for restoring communication in these patients and has attracted widespread attention^4–12^. Most BCI research to date has focused on restoring gross motor functions, such as grasping and reaching^7,13,14^, or on point-and-click typing via a computer cursor^15,16^. However, restoring fine and rapid behaviors such as handwriting could enable more efficient communication, thereby enhancing patients’ interaction abilities and quality of life^4,11,12^. Willett *et al*.^4^ were the first to decode handwriting motor intentions using an intracortical BCI, achieving a communication rate of 90 characters per minute in patients with spinal cord injury by classifying 26 letters and 5 special symbols. This breakthrough demonstrated the significant potential of handwriting BCI in enabling efficient and natural communication. Subsequently, Yang *et al*.^11^ decoded the neural representations of multi-stroke Chinese characters via an intracortical BCI, achieving 86% recognition accuracy for 3,500 commonly used characters (average 10.3 strokes per character). System performance remained stable over five months, demonstrating both broad adaptability and long-term reliability. This advancement extends handwriting BCI from linear alphabets to two-dimensionally structured scripts (Chinese characters), providing a feasible foundation for cross-linguistic restoration of fine-grained writing^12^.

However, invasive BCI based on intracortical electrodes faces substantial practical hurdles: acquiring high-quality signals requires brain surgery, and the long-term stability of implanted electrodes is susceptible to degradation from biofouling and tissue reactions^13^. Non-invasive approaches such as electroencephalography (EEG) offer a safe and accessible alternative^17^. EEG is particularly favored in BCI applications due to its low risk, affordability, and practicality^18,19^. Nevertheless, high noise levels, considerable variability across sessions and individuals, and limited spatial resolution make efficient decoding of handwriting-related EEG signals a major challenge^20,21^. Consequently, high-quality open datasets are essential for advancing decoding algorithms.

Although substantial progress has been made in invasive handwriting imagery BCI studies^4,11,12^, non-invasive EEG-based decoding of handwriting imagery still suffers from a severe lack of data. Existing publicly available EEG-BCI datasets mainly focus on motor imagery tasks (e.g., grasping, arm extension)^22–24^ or visually evoked tasks (e.g., responses to visual stimuli)^25,26^, and none provide standardized data for the handwriting imagery paradigm. To address this gap and support the development of high-performance EEG decoding algorithms, we present the first open dataset dedicated to EEG decoding of handwriting imagery.

In this study, we adopted a paradigm-driven data acquisition strategy to achieve, for the first time, non-invasive EEG-based capture of multidimensional neural representations underlying handwriting imagery (HI). We present an EEG-based BCI dataset for HI, comprising data from 21 healthy participants collected across two independent sessions. The dataset includes two experimental paradigms designed to capture distinct neural coding features. The first paradigm required participants to imagine writing five basic strokes of Chinese characters: horizontal (一, rightward), vertical (丨, downward), left-falling (丿, left-downward curve), right-falling (㇏, right-downward curve), and turning (𠃋, turning motion). These strokes serve as fundamental units of Chinese characters and can be combined to form nearly all commonly used characters. The second paradigm involved imagining six single vowels in Hanyu Pinyin (ɑ, o, e, i, u, ü), with the first five corresponding to English vowels and ü representing a Chinese front rounded vowel. The two paradigms reflect complementary dimensions of handwriting behavior: the stroke task captures neural coding of discrete motor units, whereas the single-vowel task emphasizes continuous trajectory planning, such as the circular trajectory of ɑ. Together, these paradigms provide diverse training data for decoding algorithms, enhancing BCI accuracy and adaptability in HI tasks. The dataset comprises two classification tasks: a five-class task for Chinese character strokes and a six-class task for Pinyin vowels. As the first EEG-based HI-BCI dataset of its kind, it extends beyond existing gross motor imagery EEG datasets, representing a critical advance in multi-category fine-motor decoding. Its near-balanced gender distribution (13 males, 8 females) supports the development of fair and generalizable BCI models. Future work should validate this dataset in diverse populations (older adults, motor-impaired individuals) to broaden its applicability for handwriting imagery BCI.

## Methods

### Participants

We initially recruited 25 healthy participants for this study. Following data collection, four participants were excluded due to quality concerns, including inconsistent task performance or failure to meet the task requirements. Consequently, the final dataset comprises 21 participants (13 males, 61.9%; 8 females, 38.1%) with a median age of 25 years (range: 22–32). All participants were self-reported right-handers and held at least a bachelor’s degree, ensuring consistent comprehension of the experimental tasks. Comprehensive demographic information is summarized in Table 1. All data were anonymized, with participants identified solely by IDs from “sub-01” to “sub-25”. The excluded participant IDs were “sub-02”, “sub-04”, “sub-09”, and “sub-16”.

**Table 1:** Demographic characteristics of all participants, including participant ID, age, gender, experience with brain-computer interface (BCI), and dominant hand.

Participant ID	Age	Gender	Exp with BCI	Dominant Hand
sub-01	28	Male	No	R
sub-03	24	Male	Yes	R
sub-05	23	Male	Yes	R
sub-06	27	Male	No	R
sub-07	23	Female	No	R
sub-08	25	Male	No	R
sub-10	24	Female	No	R
sub-11	23	Male	No	R
sub-12	32	Male	Yes	R
sub-13	28	Male	Yes	R
sub-14	30	Male	Yes	R
sub-15	24	Male	No	R
sub-17	27	Female	Yes	R
sub-18	22	Male	No	R
sub-19	28	Male	No	R
sub-20	27	Male	Yes	R
sub-21	22	Female	No	R
sub-22	28	Female	No	R
sub-23	25	Female	No	R
sub-24	27	Female	No	R
sub-25	24	Female	No	R

This study was approved by the Ethics Committee of Kunming University of Science and Technology (Approval No.: KMUST-MEC-2023-026). All procedures were conducted in strict accordance with the Declaration of Helsinki (2013 revision) and China’s Measures for the Ethical Review of Biomedical Research Involving Humans. Prior to data collection, all participants provided written informed consent after receiving a comprehensive explanation of the study’s purpose and procedures, authorizing the use of their anonymized data for research purposes. Inclusion criteria were healthy individuals aged 18–60 years with no history of neurological, developmental, or speech impairments. Exclusion criteria included: (1) a history of neurological, psychiatric, or cognitive disorders; (2) any medical or physiological condition that could compromise EEG signal quality; or (3) inability to consistently comply with experimental tasks.

### Experimental paradigm

**Fig. 1:**
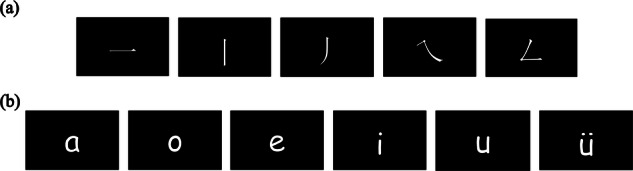
Schematic illustration of writing trajectories in the task cue phase. (**a**) Writing trajectories of the five basic strokes of Chinese characters. (**b**) Writing trajectories of Hanyu Pinyin single vowels.

This study employed two experimental paradigms designed to quantitatively capture multidimensional neural representations during handwriting imagery tasks. In the first paradigm, participants imagined writing five basic strokes of Chinese characters, with movement directions specified as follows: horizontal (一, rightward), vertical (丨, downward), left-falling (丿, left-downward arc), right-falling (㇏, right-downward arc), and turning (𠃋, turning motion). These strokes serve as discrete motor units for constructing Chinese characters. In the second paradigm, participants imagined writing six Hanyu Pinyin single-vowel symbols (ɑ, o, e, i, u, ü), with “ü” being a front rounded vowel unique to Chinese. This task required planning continuous trajectories (e.g., circular motion for “ɑ“). Each trial began with simultaneous visual and auditory instructions. During the cue phase, the writing trajectory of the target symbol (stroke or single vowel) was presented on a black background to prompt participants to initiate the HI task. In the imagery phase, the display remained black with no stimuli presented. During the rest phase, a visual cue indicated the end of the trial, also on a black background. A schematic diagram of the task cues is provided in Fig. 1. This dual-paradigm design allows for capturing distinct neural coding features: the basic stroke task emphasizes discrete motor units, while the Pinyin single-vowel task emphasizes continuous trajectory planning. Together, these paradigms provide rich, multidimensional data to train and evaluate decoding algorithms, thereby improving the accuracy and adaptability of EEG-based HI-BCI systems.

**Fig. 2:**
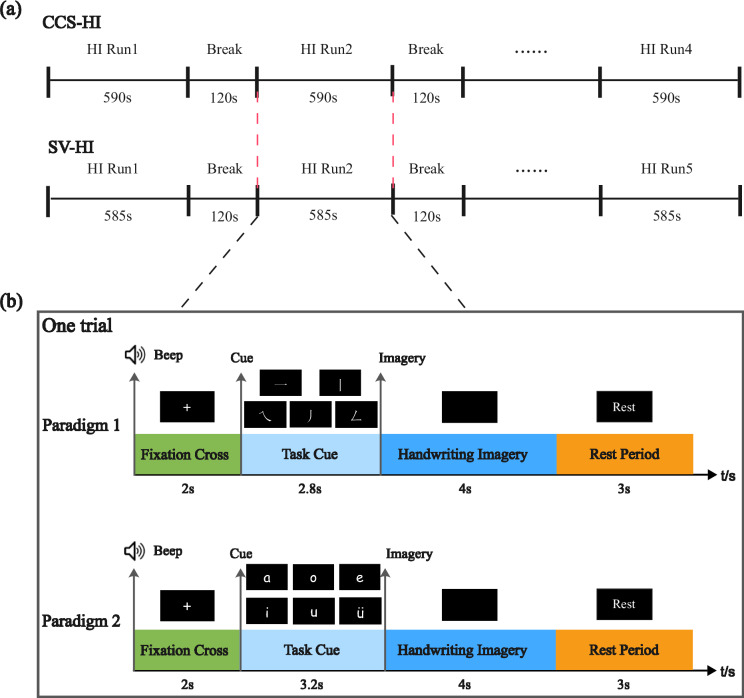
Experimental design and trial structure for handwriting imagery tasks. (**a**) Run sequence for the Chinese character stroke (CCS-HI) and Pinyin single vowel (SV-HI) tasks. (**b**) Detailed timeline of a single trial for both paradigms.

For both experimental paradigms, each participant completed two independent recording sessions on separate days (at least 24 hours apart) to evaluate inter-session and inter-participant variability. Each session lasted 45–58 minutes. The Chinese character stroke HI (CCS-HI) task comprised 4 runs of 50 trials each, while the Pinyin single vowel HI (SV-HI) task comprised 5 runs of 48 trials each (see Fig. 2a for details of the run structure). To mitigate fatigue, a mandatory 120-second rest period was provided between runs. Afterwards, participants could choose to begin the next run immediately or extend the rest. Throughout the session, task performance was monitored in real time to ensure data quality.

Figure 2b illustrates the timeline of a single trial for both experimental conditions. Each trial for the CCS-HI task lasted 11.8 s, comprising a 2-s fixation cross with a concurrent auditory cue, followed by a 2.8-s task cue phase displaying the animated trajectory of the target stroke. Participants then performed 4-s handwriting imagery, followed by a 3-s rest interval. The SV-HI task followed an identical structure, with the sole modification being an extended task cue phase (3.2 s) to accommodate the increased complexity of writing vowel symbols; all other components remained unchanged.

It is important to note that during the handwriting imagery phase, participants performed only one complete imagination of the handwriting trajectory. As shown in Fig. 2, the CCS-HI task comprised 200 trials per session, with 40 trials allocated to each Chinese character stroke type; the SV-HI task comprised 240 trials per session, with 40 trials allocated to each Pinyin single vowel type.

### Data collection & preprocessing

Prior to the experiment, participants received a detailed explanation of the experimental procedure and viewed a demonstration video of the handwriting imagery task to ensure a full understanding of the requirements. EEG data were recorded continuously using a Neuracle NeuSenW amplifier and a 32-channel Ag/AgCl electrode cap, with a sampling rate of 1000 Hz to ensure high temporal resolution. Recordings were synchronized with time-locked event markers (stored in BIDS-compliant ‘_events.tsv‘ files per session), which demarcated the onset of each 4-second motor imagery trial: each trial was part of either the CCS-HI (5 classes) or SV-HI (6 classes) task. During acquisition, electrode impedance was maintained below 10 kΩ. Electrodes were positioned according to the international 10–10 system. The detailed channel coordinates (including 3D spatial locations and nomenclature) are provided in the accompanying ‘electrodes.tsv‘ file. All equipment was professionally calibrated prior to the study to ensure data quality and reproducibility.

It is important to note that the dataset is released in its raw form, preserving the signals exactly as acquired. No preprocessing steps, such as filtering, interpolation, or normalization, were applied. Preprocessing was performed exclusively during model training and testing using the MNE-Python toolkit (full code is available in the code folder of the dataset). The preprocessing pipeline consisted of the following steps: 1) 1–40 Hz bandpass filtering using a Butterworth filter; 2) 50 Hz notch filtering to suppress power-line interference. To accurately identify anomalous channels, all EEG signals were visually inspected and manually annotated using the EEGLAB toolbox (v2023.1, MATLAB) to prevent misclassification of physiological signals by automated thresholding. Annotation information follows the BIDS specification and is documented in the respective ‘_channels.tsv’ file for each subject-session-task combination (e.g., ‘sub-05_ses-01_task-CCSHI_channels.tsv’), with the ‘bad’ field indicating anomalous channels. Non-EEG status channels (e.g., ‘Status’) were first removed to ensure that only valid EEG channels were processed. Marked bad channels were then repaired using spherical spline interpolation. The data were subsequently downsampled from 1000 Hz to 250 Hz to achieve an optimal balance between temporal resolution and computational efficiency. Finally, z-score normalization was applied to each channel of every trial to remove inter-channel amplitude differences. The preprocessed trial-level EEG data (4-second imagery epoch, 0–4 s post-cue) from both CCS-HI and SV-HI tasks were used for subsequent model training and testing.

Through rigorous verification of trial integrity, most data were confirmed to meet the preset criteria (CCS-HI: 200 trials/session, SV-HI: 240 trials/session), with two anomalies identified: For sub-06’s SV-HI task (ses-02), only 225 trials were retained due to trigger synchronization errors (a shortfall of 15 trials); for sub-13’s CCS-HI task (ses-02), 162 trials were retained due to subject fatigue (a shortfall of 38 trials). All retained trials passed the signal quality check and were included in the final dataset. The trial shortfalls were evenly distributed across task categories (maximum deviation ≤ 2 trials, well below the 5% imbalance threshold), so category balancing measures for model training were unnecessary.

## Data Records

Data collected in this study are freely accessible via the figshare platform^27^. As a general data repository, figshare ensures the public availability of research outputs in a citable, shareable, and discoverable format. Both source and metadata files within this dataset follow the EEG-BIDS specification^28^.

**Fig. 3:**
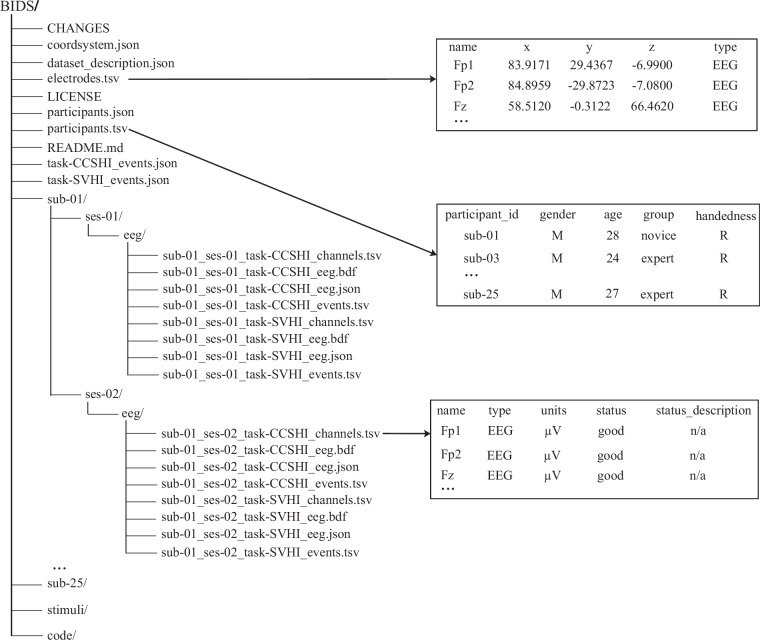
BIDS-compliant hierarchical structure of the EEG dataset. Data are organized by participant (sub-<label>), session (ses-<label>), and eeg/ folder. Associated metadata files include electrode coordinates, channel quality, and participant demographics, supporting automated analysis pipelines and reproducible research.

Figure 3 presents the BIDS-compliant directory structure of the EEG dataset along with a representative preview of the data. The figure illustrates in detail only the data structure of a single participant (‘sub-01’), while other participants follow the same hierarchical organization to avoid redundancy. Data are organized by participant (‘sub-<label>‘), session (‘ses-<label>‘), and ‘eeg/‘ folder. Metadata stored in the root directory define the scope of the dataset, the experimental design, and technical parameters. The core files include the global description file ‘dataset_description.json’, specifying dataset name, license, BIDS version, and acquisition system; the participant attribute file ‘participants.tsv’, containing demographic information such as age, gender, and handedness, as well as phenotypic characteristics such as proficiency (novice/expert); electrode coordinate files ‘coordsystem.json’ (defining the EEG electrode coordinate system) and ‘electrodes.tsv’ (providing 3D coordinates in millimeters for 32 electrodes according to the International 10–10 System); and task event specification files ‘task-CCSHI_events.json’ and ‘task-SVHI_events.json’, defining event field metadata for the CCS-HI and SV-HI tasks, respectively. This organization and the accompanying metadata enable automated analysis and support reproducible research.

In addition, the dataset comprises 21 subject folders (e.g., ‘sub-01’, ‘sub-25’), corresponding to the 21 participants ultimately included in the analyses. Within each ‘session’ folder (‘ses-01’, ‘ses-02’), the ‘eeg/‘ subfolders are organized by task (CCS-HI, SV-HI) and contain four core file types: _channels.tsv, which records channel metadata such as channel name, type (EEG), and status (good/bad channel); _eeg.bdf, which stores raw EEG data in Neuracle native format, including 32-channel voltage signals (unit: μV) and hardware-triggered event markers; _eeg.json, which describes EEG acquisition parameters (e.g., 1000 Hz sampling rate and reference electrodes); and _events.tsv, which logs task-related event information (including onset in seconds, duration, and trial_type) for alignment of EEG data with behavioral performance. Supplementary resources include the ‘stimuli’ folder, containing the visual stimuli used for the tasks, and the ‘code’ folder, which provides Jupyter notebooks (e.g., ‘CCSHI_EEGNet.ipynb’) and Python scripts (e.g., ‘Covert_Event.py’) for preprocessing, model training, and visualization. Instructions for using these code resources are detailed in the Code Availability section.

## Technical Validation

Classification Performance. To verify the distinguishability of different task conditions in handwriting imagery EEG signals, we used EEGNet^29^—a deep learning framework widely adopted in EEG decoding—to evaluate classification performance. Specifically designed to process the spatiotemporal features of EEG signals, this model exhibits excellent adaptability in neural decoding for tasks such as motor intention and language cognition, enabling effective capture of task-specific patterns. Its open-source nature has further facilitated widespread application across EEG-BCI studies. As a compact convolutional neural network (CNN), it synchronously optimizes spatial channel weights and temporal dynamic features via depthwise separable convolution; its spatiotemporal hybrid extraction mechanism is highly compatible with the brain-region specificity of handwriting imagery and the neural oscillations associated with handwriting trajectories. Moreover, the model’s ability to perform well with limited training data aligns with typical EEG-BCI research requirements.

When evaluating the classification performance of handwriting imagery EEG signals, we employed a rigorous approach to ensure robust training and unbiased results. Five-fold cross-validation was implemented on the training set (Session 1), with reproducible data partitioning ensured by a fixed random seed (seed = 42). For each fold, data were split into training and validation sets in an 8:2 ratio, while preserving the original trial distribution across all task categories. Models were trained using the Adam optimizer with a learning rate of 0.0001—chosen to mitigate parameter oscillation and enhance convergence on small EEG datasets—and a weight decay of 0.08 for L2 regularization to suppress overfitting. Cross-entropy loss was adopted as the optimization objective for this multi-classification task. During training, loss and accuracy dynamics were monitored on both the training and validation sets to evaluate model fitting, generalization, and overfitting risk in real time. To further simulate the cross-session generalization challenges inherent in real-world BCI systems, trained models were directly evaluated on the independent Session 2 dataset (collected ≥ 24 hours apart). This two-tier evaluation framework—cross-validation on Session 1 followed by independent testing on Session 2—leverages the compact architecture of EEGNet to maximize data efficiency, rigorously assesses robustness to spatiotemporal noise, and closely mirrors dynamic interference scenarios in practical BCI, thereby ensuring the model’s applicability in real-world settings.

**Fig. 4:**
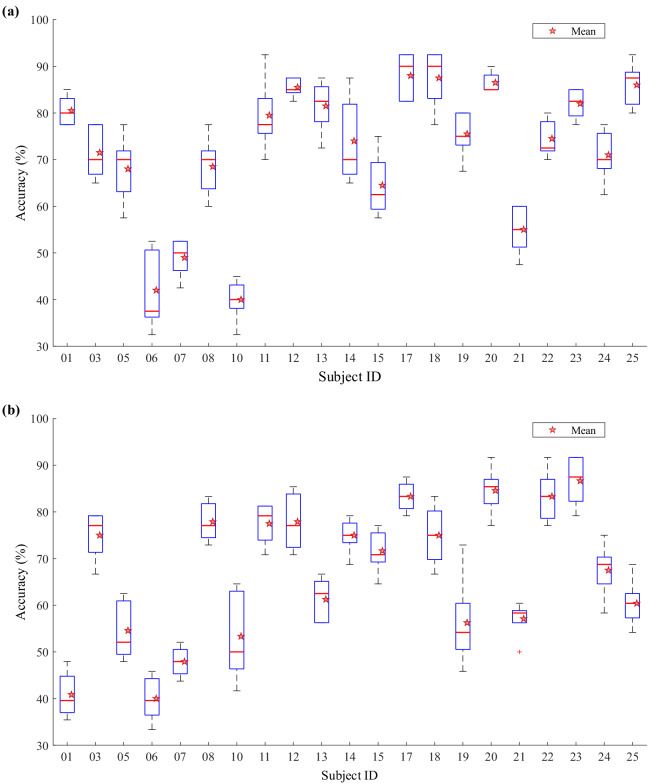
Classification accuracy distributions from 5-fold cross-validation in Session 1 for 21 participants. Boxplots: per-participant accuracy distributions across folds; asterisks: per-participant mean accuracy. (**a**) CCS-HI task. (**b**) SV-HI task.

The distribution of classification accuracies from the 5-fold cross-validation within Session 1 (under the aforementioned evaluation framework) is illustrated in Fig. 4. This figure shows the accuracy distributions of 21 participants in the CCS-HI task (Fig. 4a, Chinese character stroke handwriting imagery task) and the SV-HI task (Fig. 4b, Pinyin single-vowel handwriting imagery task). For each participant, the asterisk denotes their mean accuracy averaged across the five cross-validation folds. The overall mean accuracy for the CCS-HI task (>70%) was higher than that for the SV-HI task. Both tasks exhibited notable individual differences: participants sub-06, sub-07, and sub-10 showed consistently lower accuracies in both tasks (with the lowest mean around 40%). For the CCS-HI task (a 5-class task), the remaining participants achieved mean accuracies above 60%, significantly exceeding the chance level (20%). Similarly, for the SV-HI task (a 6-class task), the remaining participants achieved mean accuracies above 50%, significantly exceeding the chance level (≈16.67%). Additionally, several participants reached average classification accuracies above 80% across both tasks. These results suggest that the neural features associated with Chinese character stroke and Pinyin single-vowel handwriting imagery are reliably distinguishable in EEG signals.

Although classification performance within the same session (Table 2) validates the discriminability of task-related neural features, cross-session generalization ability better reflects the stability of neural features over time. As shown in Table 2, the average cross-session accuracy for CCS-HI was 63.06%, which was significantly higher than the 5-class chance level (20%; *t*(20) = 13.21, *p* < 0.001). For SV-HI, the average cross-session accuracy was 60.14%, significantly exceeding the 6-class chance level (16.67%; *t*(20) = 13.63, *p < *0.001). Further individual-level analyses revealed remarkable consistency in performance across sessions for both tasks. Among high-performing participants (e.g., sub-12, sub-17, sub-20), cross-session accuracy closely matched their within-session performance: all achieved cross-session accuracies of ≥82.00% in the CCS-HI task (e.g., sub-20 maintained a cross-session accuracy of 82.00% despite a within-session accuracy of 86.50%), and ≥77.08% in the SV-HI task. In contrast, low-performing participants (e.g., sub-06, sub-07) exhibited persistently poor cross-session performance in both tasks, with accuracies ≤ 40.00% for CCS-HI and ≤ 41.25% for SV-HI. Group-level analyses further confirmed this pattern: in the CCS-HI task, cross-session accuracy across the 21 participants was strongly positively correlated with within-session accuracy (*r* = 0.83, 95% CI [0.62, 0.93], *t*(19) = 6.46, *p* < 0.001). This correlation was also significant for the SV-HI task (*r = *0.80, 95% CI [0.57, 0.92], *t*(19) = 5.83, *p* < 0.001). These individual differentiation patterns—particularly the stability among extreme performers and the robust correlations at the group level—are highly consistent across both tasks. Together, these findings demonstrate that the neural representations underlying Chinese character stroke and Pinyin single-vowel handwriting imagery exhibit robust cross-session stability, supporting their potential for reliable BCI applications.

**Table 2:** Within-session and cross-session classification accuracy for CCS-HI and SV-HI handwriting imagery tasks.

Subject	CCS-HI (Within)	CCS-HI (Cross)	SV-HI (Within)	SV-HI (Cross)
sub-01	80.50	62.00	40.83	38.33
sub-03	71.50	66.00	75.00	60.00
sub-05	68.00	39.00	54.58	41.25
sub-06	42.00	40.00	40.50	38.67
sub-07	49.00	40.00	47.92	31.25
sub-08	68.50	74.50	77.92	74.58
sub-10	40.00	43.50	53.33	60.83
sub-11	79.50	79.50	77.50	66.25
sub-12	85.50	74.50	77.92	82.92
sub-13	81.50	64.20	61.25	60.42
sub-14	74.00	59.00	75.00	74.17
sub-15	64.50	50.50	67.08	57.92
sub-17	88.00	83.50	83.33	80.00
sub-18	87.50	71.50	75.00	55.42
sub-19	75.50	73.50	56.25	57.50
sub-20	86.50	82.00	84.58	77.08
sub-21	55.00	46.50	57.08	55.00
sub-22	74.50	81.00	83.33	67.08
sub-23	82.00	74.00	86.67	77.50
sub-24	71.00	56.00	67.50	53.75
sub-25	86.00	63.50	60.42	52.92
mean	71.93	63.06	66.81	60.14
std	14.58	14.94	14.41	14.62

Note: Within-session classification accuracy refers to the average result of 5-fold cross-validation.

To evaluate generalization across sessions, Table 2 compares the within-session accuracy obtained from 5-fold cross-validation on Session 1 data with the cross-session accuracy achieved when a classifier trained on Session 1 was tested on Session 2. A consistent performance drop was observed: for CCS-HI, accuracy decreased by 8.87 percentage points from 71.93% (Session 1) to 63.06% (Session 2); for SV-HI, it decreased by 6.67 percentage points from 66.81% to 60.14%. Notably, cross-session decoding accuracy was significantly higher for CCS-HI than for SV-HI (63.06% vs. 60.14%, *t*(20) = 2.76, *p* = 0.012, paired *t*-test). This advantage likely stems from fundamental disparities in motor encoding: discrete, overlearned Chinese character strokes—acquired through years of repetitive handwriting practice—elicit robust and consistent sensorimotor activation patterns, whereas Pinyin vowel gestures involve continuous, abstract trajectories (e.g., the circular ‘a’ or hooked ‘u’) that rely on dynamic cognitive-motor integration, making them more susceptible to mental-state fluctuations across sessions. Consistent with the reviewer’s observation, participants with prior BCI experience (*n* = 7) achieved significantly higher cross-session decoding accuracy than naïve users (*n* = 14) in both tasks—CCS-HI: 66.9% vs. 61.1% (*p* = 0.008); SV-HI: 68.0% vs. 56.4% (*p* = 0.003)—suggesting that user expertise enhances longitudinal BCI reliability.

**Fig. 5:**
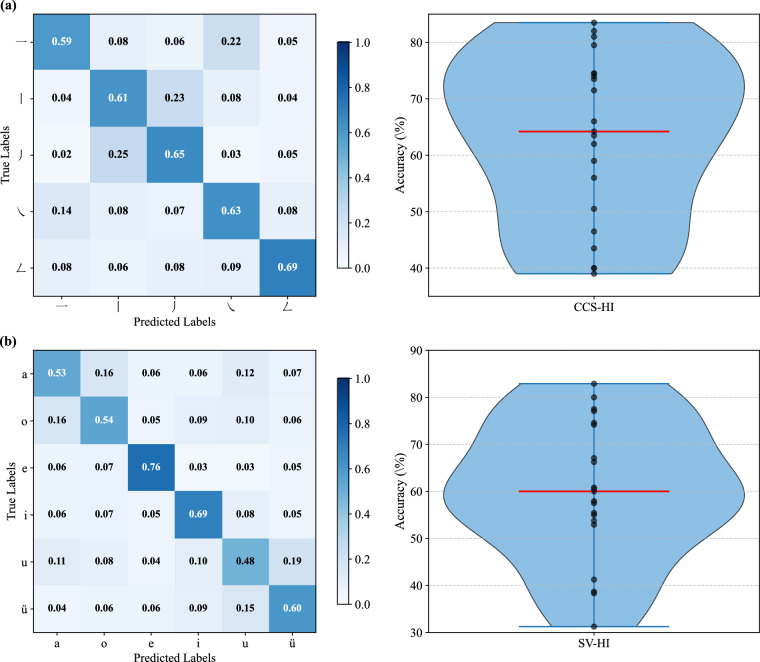
Visualization of cross-session classification performance. (**a**) CCS-HI task: averaged confusion matrix (left, *N* = 21) and participant-level accuracy distribution (right). (**b**) SV-HI task: averaged confusion matrix (left, *N* = 21) and participant-level accuracy distribution (right). For both confusion matrices, diagonal elements represent per-class accuracy. For the violin plots (right panels), the red line indicates the median, black dots denote individual participant accuracies, and whiskers show the full data range.

To further visualize error patterns and individual differences in cross-session decoding, Fig. 5 presents the average confusion matrices and participant-level accuracy distributions on the independent test set (Session 2) for both CCS-HI and SV-HI tasks across all 21 participants. In the CCS-HI task, the confusion matrix revealed task-specific inter-class interference patterns: the horizontal (“一”) and right-falling (“㇏”) strokes showed a mutual confusion rate of 0.22, likely due to shared neural representations underlying horizontal movement imagery. The left-falling (“丿”) and vertical (“丨”) strokes had a confusion rate of 0.25, potentially reflecting spatial-semantic similarities in their downward kinematic trajectories, specifically the shared vertical component. In contrast, the turning stroke (“𠃋”) achieved the highest class-specific accuracy (0.69). Its unique directional-turn trajectory produces distinct spatiotemporal dynamics, forming robust task-specific neural signatures that reduce confusion with other strokes.

For the SV-HI task, confusions were concentrated among Pinyin single vowels with similar handwriting trajectory features: the confusion rate between ‘a’ and ‘o’ was 0.16, as both have rounded, curved trajectories (similar morphology); the confusion rate between ‘u’ and ‘ü’ was 0.19, due to their shared movement pattern—both start with a vertical hook followed by a lateral extension. This suggests that the neural representations of motor intentions underlying their handwriting imagery are highly similar, potentially arising from cross-activation and overlap at the encoding level. In contrast, ‘e’ (accuracy = 0.76) and ‘i’ (accuracy = 0.69) were more easily distinguishable due to their unique handwriting trajectories: the horizontal folded trajectory of ‘e’ differs distinctly from the rounded curves of ‘a’ and ‘o’; meanwhile, the simple trajectory of ‘i’—dominated by a vertical stroke with a dot—stands in sharp contrast to the complex hooked extension movements of ‘u’ and ‘ü’. These unique motor features likely produce more distinct neural representations, thereby resulting in higher classification accuracy.

To further characterize the distribution pattern of individual decoding performance, Fig. 5 also presents violin plots of participant-level accuracy. For the CCS-HI task, accuracy exhibited a negatively skewed distribution (mean = 63.06%, median = 64.20%), with the highest density in the mid-to-high accuracy range (50–80%) and a sparse left tail extending down to approximately 40%, indicating that most participants achieved mid-to-high performance. In contrast, the SV-HI task displayed an approximately symmetrical, unimodal distribution (median ≈ 60%) with an extended lower tail reaching down to 30%, suggesting that confusion among certain single-vowel pairs (e.g., “a”/“o”, “u”/“ü”) adversely affected only a subset of participants. Notably, both tasks contained a high-performance subgroup (accuracy: CCS-HI ≥ 80%, SV-HI ≥ 77%), manifested as upper-end data points in the distributions. Crucially, the overall accuracy of this subgroup exceeded that of the most distinguishable individual categories in the confusion matrices (e.g., turning stroke “𠃋”: 69%; vowels “e” and “i”: 76% and 69%, respectively). This indicates that these participants achieved superior decoding performance not merely by excelling on a few easy classes, but by maintaining high accuracy across all classes, including those commonly confused. From the perspective of individual differences, these results demonstrate that task-specific neural features are stably expressed in most participants, albeit modulated by inter-class confusion. Moreover, high-performing individuals appear to possess an enhanced capacity to resolve these common confusions.

### Neural correlates of performance variation

**Fig. 6:**
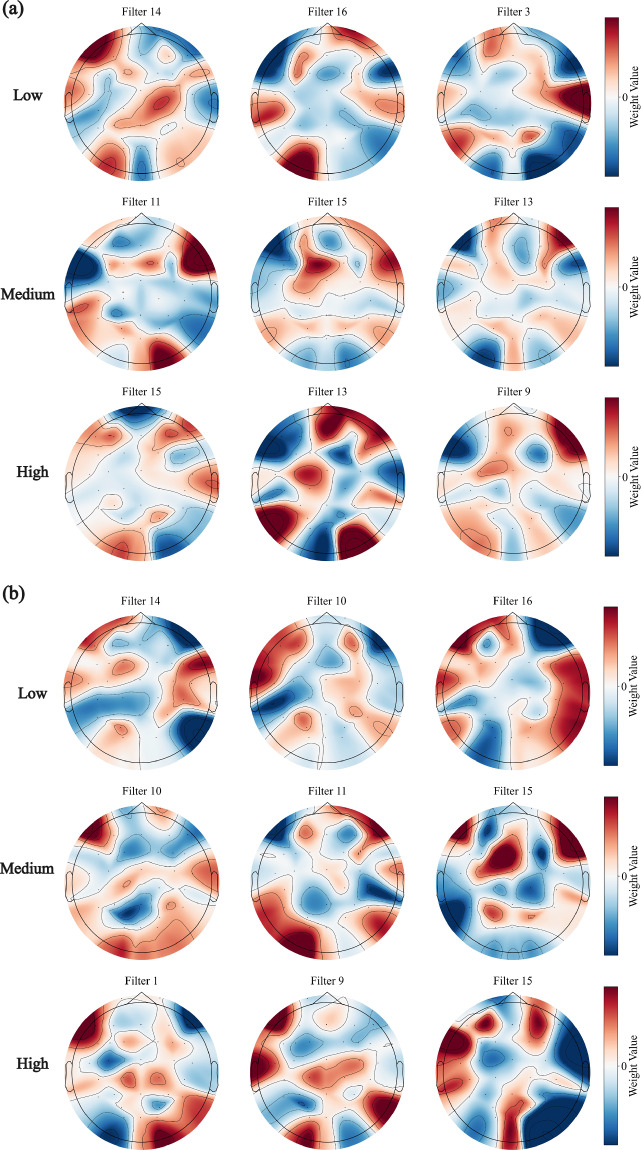
Topographic maps of EEGNet spatial filters, stratified by subject performance. Based on cross-session classification accuracy, the top three spatial filters (K1, K2, K3) with the highest variance across channels are shown for each performance group, where (**a**) corresponds to the Chinese character stroke (CCS-HI) task and (**b**) to the Pinyin single vowel (SV-HI) task.

Analysis of EEGNet spatial filters revealed task-specific differences in brain region weight distributions (Fig. 6). For the CCS-HI task (Fig. 6a), spatial filters (e.g., Filter 14, 16) showed high-weight (red regions) localization predominantly over the left sensorimotor cortex—this pattern aligns with the neural substrates of discrete single-stroke handwriting imagery (the core of Chinese character stroke tasks). Even when participants mentally simulate only the writing trajectory without explicitly imagining hand movement, the internal motor representation of writing in right-handed individuals typically exhibits left-hemispheric dominance, reflecting the lateralized organization of skilled hand motor programs. In contrast, for the SV-HI task (Fig. 6b), filters (e.g., Filter 10, 16) exhibited broad high-weight distributions across central-parietal and bilateral temporal regions; this widespread localization reflects the increased demands of continuous multi-stroke handwriting imagery and visuospatial processing required to mentally generate the complex shapes of Latin-script vowel letters (e.g., “a”, “o”), which involve trajectory sequencing, curvature control, and spatial layout planning—functions typically supported by parietal-temporal networks.

More critically, within the same task, there is a systematic pattern separation between different performance groups: the filter topologies of high-performance participants are concentrated and stable, while those of low-performance participants show scattered and more heterogeneous distributions. Quantitative analyses further showed that filter weight variance was negatively correlated with cross-session classification accuracy. For example, in the CCS-HI task, the high-performance group exhibited a focused weighting pattern in the left motor cortex (C3/FC3), whereas the low-performance group had a diffuse distribution extending into non-primary motor regions; in the SV-HI task, the high-performance group showed a more stable weight distribution in the central-parietal and temporal regions.

These results indicate that differences in decoding performance arise from the focus and stability of spatial neural representations. High-performance participants exhibit neural patterns that are more concentrated in task-relevant brain regions, enabling the model to extract robust and consistent features. In contrast, low-performance participants display more diffuse patterns, potentially introducing additional noise and interference. Collectively, these findings not only elucidate the neural mechanisms underlying inter-individual variability in decoding performance but also highlight potential targets (e.g., task-specific brain region focus) for individualized model optimization and neurofeedback training in BCI applications.

## Usage Notes

This dataset comprises two core tasks: CCS-HI (Chinese Character Stroke Handwriting Imagery) and SV-HI (Pinyin Simple Vowel Handwriting Imagery). The raw EEG data are available from the Figshare repository (10.6084/m9.figshare.29987758.v4). Users should note the following data integrity considerations:

- For sub-06, the SV-HI task (ses-02) contains 225 trials due to a trigger synchronization error, resulting in 15 fewer trials than the preset 240.
- For sub-13, the CCS-HI task (ses-02) contains 162 trials due to participant fatigue, representing a shortfall of 38 trials from the preset 200.

It should be noted that the above-mentioned trial shortfalls were evenly distributed across task categories (maximum inter-category deviation ≤ 2 trials), and the imbalance rate across categories did not exceed 5%. Thus, no additional category balancing measures are required.

For loading and analyzing EEG data, users can use the EEGLAB toolbox (https://sccn.ucsd.edu/eeglab) in MATLAB or the MNE-Python library (https://mne.tools) in Python. For decoding, the scikit-learn (https://scikit-learn.org/stable) and PyTorch (https://pytorch.org/) libraries are employed; the corresponding code is available in the ‘code’ directory of this repository. The code has been validated in environments with scikit-learn 1.6.1, PyTorch 2.0.0 + cu117, and MNE-Python 1.7.0. Detailed instructions on environment setup, script workflow, and usage can be found in the README file within the ‘code’ folder.

## Data Availability

The EEG dataset generated and analyzed in this study is publicly available on figshare (10.6084/m9.figshare.29987758.v4). The data are structured in compliance with the EEG-BIDS standard, featuring participant- and session-level directories alongside detailed metadata (e.g., data description, participant information, electrode locations, and event markers) to support reproducible research. Additional resources, including experimental stimuli and custom code for data preprocessing, model training, and visualization, are available within the same repository.

## Code availability

All code scripts used in this study are publicly available in the same figshare repository as the dataset (10.6084/m9.figshare.29987758.v4), under the code/ directory. Users can locate the code by scrolling through the online file list or downloading the full repository and navigating to /code. Detailed setup instructions—including mappings to the original BIDS format—are provided in code/README.md.
